# How Do Orodispersible Tablets Behave in an In Vitro Oral Cavity Model: A Pilot Study

**DOI:** 10.3390/pharmaceutics12070651

**Published:** 2020-07-09

**Authors:** Neel Desai, Andrew Redfearn, Graeme MacLeod, Catherine Tuleu, Ben Hanson, Mine Orlu

**Affiliations:** 1 Department of Pharmaceutics, School of Pharmacy, University College London, London WC1N 1AX, UK; neel.desai.13@ucl.ac.uk (N.D.); agredfearn@gmail.com (A.R.); c.tuleu@ucl.ac.uk (C.T.); 2 SPI Pharma Inc., 503 Carr Road, Wilmington, DE 19809, USA; GMacleod@spipharma.com; 3 Department of Mechanical Engineering, Roberts Engineering Building, University College London, London WC1E 7JE, UK

**Keywords:** acceptability, age-appropriate formulation, disintegration testing, dosage form design, orodispersible tablets, test methods for new dosage forms

## Abstract

Orodispersible tablets (ODTs) offer rapid disintegration of the dosage form when placed on the tongue, which leads to fast release of the active pharmaceutical ingredient. Despite increased use in diverse patient populations, there have been numerous challenges associated with ODTs. One such concern is the lack of standardised assessment of disintegration behaviour. In the European Pharmacopoeia, ‘orodispersibles’ are defined as such if disintegration time is faster than 3 min. Common in vitro measurement methods only provide single time point data and have limited physiological accuracy. To determine more bio-predictive disintegration kinetics, a bench-top in vitro oral cavity model (OCM) was modified and piloted to assess disintegration of three ODTs of differing hardness. All ODTs disintegrated similarly within the OCM—surface breakdown/swelling, initial ‘wash away’ and final ‘wash away’. The distinct advantage presented within this pilot study using the OCM is the opportunity to ascertain disintegration behaviour profiles of ODTs by evaluating changes in the observable area during simulated oral processing. The model could be implemented as a decision-support tool during the early stages of the drug design process to improve acceptability and further understand ODT disintegration behaviour.

## 1. Introduction

Orodispersible tablets (ODTs) offer, among other advantages, rapid disintegration of the dosage form when placed on the tongue, which leads to fast release of the active pharmaceutical ingredient. This has led to the manufacture and licensing of ODTs to improve the effectiveness of treatment in numerous clinical conditions such as attention deficit hyperactivity disorder (ADHD) [1,2], epilepsy [3], pain [4], and psychiatric illnesses [5].

The flexibility offered in administration (either pre-dispersed in a suitable vehicle, dispersed directly in the mouth, or swallowed whole [6]) allow ODTs to improve patient compliance and acceptability when compared to conventional tablets. ODTs are generally favoured in paediatric [7] and geriatric [8] patient populations due to taste, texture, ease of use, and reduced concerns for difficulties in swallowing [9,10].

Despite increased prevalence and use in diverse patient populations, there have been numerous challenges associated with ODTs. Of particular concern is the lack of a standardised method for assessing disintegration time and behaviour. In the European Pharmacopoeia, dosage forms are defined as ‘orodispersible’ if disintegration time is faster than 3 min [11] when using the specified methodology and in vitro apparatus. This commonly used test provides only a single time point measure. Additionally, the assessment is far from the physiological processes that commonly occur within the oral cavity. Reasons include a lack of physiologically relevant media, which exhibits dosage forms to substantial fluid volumes and imprecise pressures as well as the absence of any information on disintegration kinetics over time.

A number of other methods have been proposed to simulate ODT disintegration including the Petri dish method whereby disintegration time is visually observed [12], the texture analyser whereby disintegration time is estimated from a force-displacement profile [13], or more novel techniques, such as the implementation of a rotary shaft to exert mechanical pressure [14]. These in vitro disintegration methods only determine the time required for orodispersibles to break down, which are without the ability to analyse physiological and mechanical forces exerted onto orodispersibles in the oral cavity. This study recommends the use of a purpose-built experimental apparatus to determine the disintegration kinetics of ODTs during oral processing, which is necessary for accurate in vivo predictions.

To ascertain these more bio-predictive disintegration kinetics, a bench-top in vitro oral cavity model (OCM), previously developed to assess the flow properties of non-Newtonian fluids during swallowing [15], and modified to study orodispersible films (ODFs) under biorelevant mechanical stimulation and synthetic saliva interactions [16] was used.

The model comprises of a soft silicone body and fixed acrylic plate mimicking the human tongue and hard palate surfaces. During a simulated swallow, the ‘tongue’ moves upwards and experiences a controlled compression onto the acrylic plate. This mimics swallowing motions observed in vivo where the tongue applies a pressure wave to the hard palate, which moves from the anterior to the posterior [17].

The aim of the study was to adapt further and pilot the use of the novel in vitro oral cavity model to examine the disintegration behaviour of three ODTs each with a different hardness under more biorelevant conditions. Additionally, the ODTs were assessed using the European Pharmacopoeia defined methodology and apparatus to determine the single disintegration time point of the ODTs using the common accelerated test.

## 2. Materials and Methodology

### Materials

Three ODTs compressed at 5, 10, and 15 kN with hardness of 1.9, 8.1, and 13.3 kilopond (kp) were supplied by SPI Pharma (Wilmington, DE, USA). Each were placebo tablets formulated with Pharmaburst^®^ 500, an ODT platform comprising highly soluble components, and co-processed to improve internal porosity, which enables quick liquid penetration into the tablet matrix [18], a 2% *w*/*w* red colouring agent that allows for visualisation within the OCM and 2.5% Lubripharm as lubricant. The supplied ODTs had a mean mass of 350 mg (±2.3 mg) with average dimensions of an 11-mm diameter by 4-mm height. The composition and preparation of simulated salivary fluid (SSF) was completed according to the formulation reported by Gittings et al. [19].

## 3. Disintegration Testing Using the Oral Cavity Model

The in vitro OCM is an electromechanical device that simulates oral conditions during swallowing [15]. A trapezoidal silicone tongue (70 × 60 mm) sat within the OCM. Dimensions and shape were chosen based on previously published studies of human anatomy using ultrasound [20,21], magnetic resonance imaging [22], and clinical video-fluoroscopic swallow studies [17,23,24]. Since the tongue is an active muscle, the physiological material properties are complex and variable. Nonetheless, published reference data are available for the Young’s modulus of human tongue in a tensed state: 125 ± 55 kPa, [25,26]. In this model, a porous structure of silicone (Smooth-On, Inc., Macungie, PA, USA, E = 370 kPa) was used to achieve the specific Young’s modulus (132 kPa) necessary for the model tongue. The OCM was stored in a temperature-controlled environment (20 °C) to ensure continuity between sample assessments.

**Figure 1:**
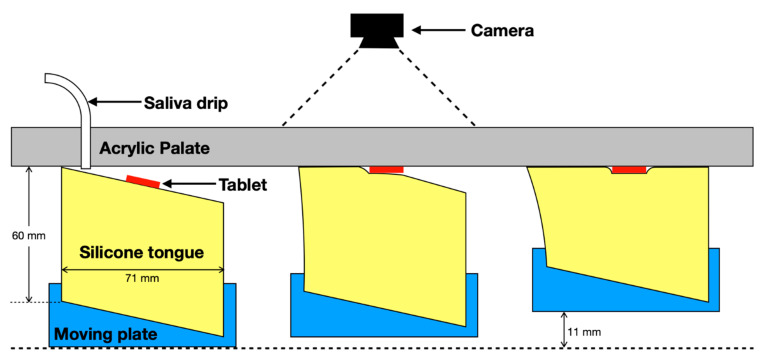
Schematic of oral cavity model (OCM) (side view/sagittal plane). On the left is the initial position and on the right is full compression.

The OCM was re-programmed from the previous study [16] to perform regular compressions of the compliant silicone tongue against the hard palate. This motion simulated typical oral manipulation. A controller (Arduino Uno, Aurdino, Italy) was used to program compression sequences, which resulted in the base of the tongue rising 11 mm. This causes an anterior–posterior sweeping pattern between the tongue and palate. Each ODT sample was placed at the median position of the surface of the compliant silicone tongue and the compression sequence was initiated (Figure 1). During compression cycles, the silicone tongue applied pressure to the ODT, which increased from 0–30 kPA at the median section of the palate [27]. The cavity was irrigated continuously with SSF, stored in the same temperature-controlled environment as the model, at a rate of 1.5 mL/min, introduced through a syringe driver [28]. As a result, a thin layer of SSF formed on the tongue surface with a continuous flow down the tongue in the anterior to posterior direction. Visual measurements of ODT disintegration within the cavity allowed for disintegration-time profiles to be established, which is an improvement on current in vitro tests that report a single degradation time [16].

**Figure 2:**
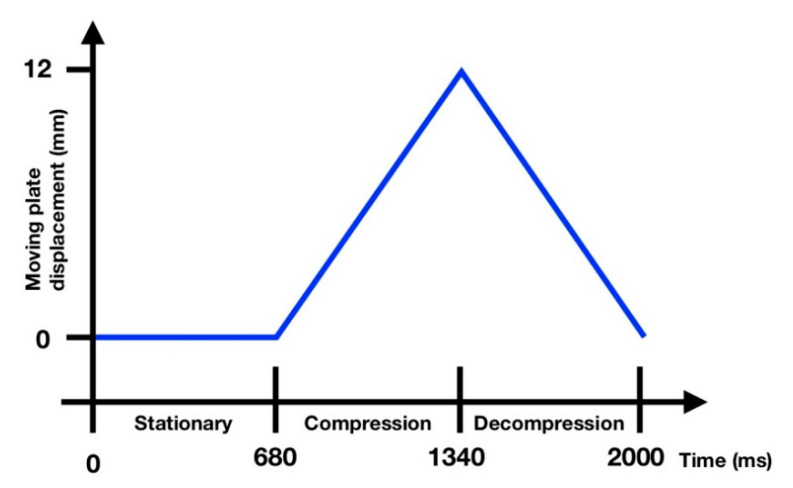
Moving plate displacement during a compression cycle. The cycle is broken up into three phases, stationary, compression, and decompression.

A mobile phone (Apple iPhone X, Apple Inc., Cupertino, CA, USA) recording at 30 frames per second (fps) was positioned above the acrylic plate facing downwards. The moving plate was raised vertically at a constant speed (18 mm/s). Vertical displacement stopped when the anterior end of the tongue was in contact with the palate. Decompression followed and the tongue returned back to the starting position with subsequent 680-millisecond pause. The compression sequence (Figure 2) lasted two seconds and was repeated until the ODT had completely disintegrated, at which point, the time was recorded (*note*: complete disintegration was an observed feature during OCM testing).

An image analysis procedure was developed and written using MATLAB (MathWorks, Natick, MA, USA). From the original video files, a single frame was extracted between the compression and decompression phases (Figure 2, 1340 ms). An edge detection method was used to identify the perimeter of the ODT during disintegration from which the area (in mm^2^) could be derived. The tablet area for each frame was plotted against time to show disintegration-time profiles for each ODT tested. Testing was carried out six times (*n* = 6) for each ODT type with mean and standard deviation areas calculated using built-in MATLAB functions.

## 4. Disintegration Testing Using the European Pharmacopoeia Methodology

Each ODT type was also assessed using testing conditions and apparatus specified in the European Pharmacopoeia [11] to determine the single disintegration time point, which is briefly outlined as follows. A single ODT was placed into three of the six cylinders of the basket-rack assembly in which each is filled with 900 mL of distilled water with discs added. The disintegration apparatus was operated so that the baskets were raised and lowered at a constant frequency (31 cycles per minute) and distance (54 mm) into a water bath, and thermostatically maintained at 37 °C. The time point at which complete disintegration was observed for each ODT was recorded. Each ODT type was tested six times (*n* = 6) with mean and standard deviation times calculated using Origin Pro (Origin Corporation, Northampton, MA, USA). To be considered a pass, each ODT type must have achieved complete disintegration inside of three minutes [11].

## 5. Results and Discussion

Table 1 displays the mean disintegration endpoint times with respective standard deviations (*n* = 6) for the ODTs tested in the OCM and European Pharmacopoeia apparatus.

## 6. Disintegration Testing Using the European Pharmacopoeia Methodology

When ODTs were tested using the European Pharmacopoeia method, all three types achieved complete disintegration far within the 180-s cut-off limit. It is unsurprising that, if the stricter criterion outlined in the US Pharmacopoeia were observed, where there is an in vitro disintegration time limit of 30 s or less [29], the tablets which were highest in hardness (13.3 kp represents ODT upper limit) would fail. This demonstrates the narrow window of design space for the formulator, which does not necessarily relate to in vivo/patient needs.

ODT disintegration was related to the materials used in the formulation. Studies have shown Pharmaburst absorbs significantly more fluid than other ODT systems, which achieves rapid disintegration [12]. The observed differences in disintegration time seen with this methodology are likely due to changes in ODT hardness, as all three had equal composition. The harder the ODT, the higher the density and lower the porosity. The mechanical stress applied by the added discs are likely to have accelerated disintegration time for all ODT types. A previous study reported that force applied during disintegration with a traditional apparatus on tablets was 0.19 N and force on the tablet with a disc added was 0.16 N. When using the disc alone, the force was observed to be far greater (0.31 N) [30]. A single time measurement does not provide a complete overview of disintegration. Monitoring changes in disintegration mechanism profiles for any pharmaceutical dosage form is likely to provide greater insight.

## 7. Disintegration Testing Using the Oral Cavity Model

**Figure 3:**
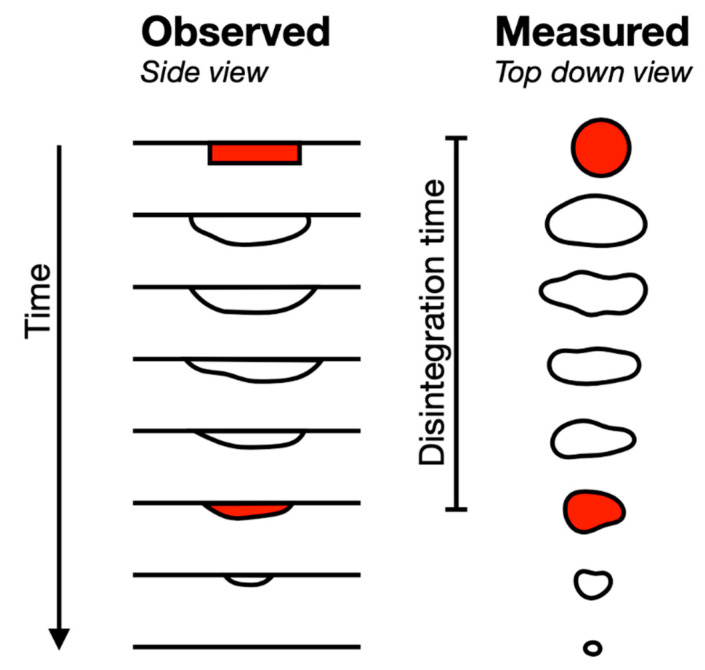
Observed (left) vs. measured (right) Orodispersible tablets (ODT) disintegration. Note: the measured ‘disintegration time’, which is the time taken to reach an area equal to starting area, was used to calculate mean disintegration time of the ODTs.

When ODTs were examined using the OCM, the disintegration times observed were significantly longer compared to the same samples assessed by the previously mentioned in vitro method. Furthermore, the observed and measured performance of the ODTs in the OCM differed during the simulation (Figure 3).

**Figure 4:**
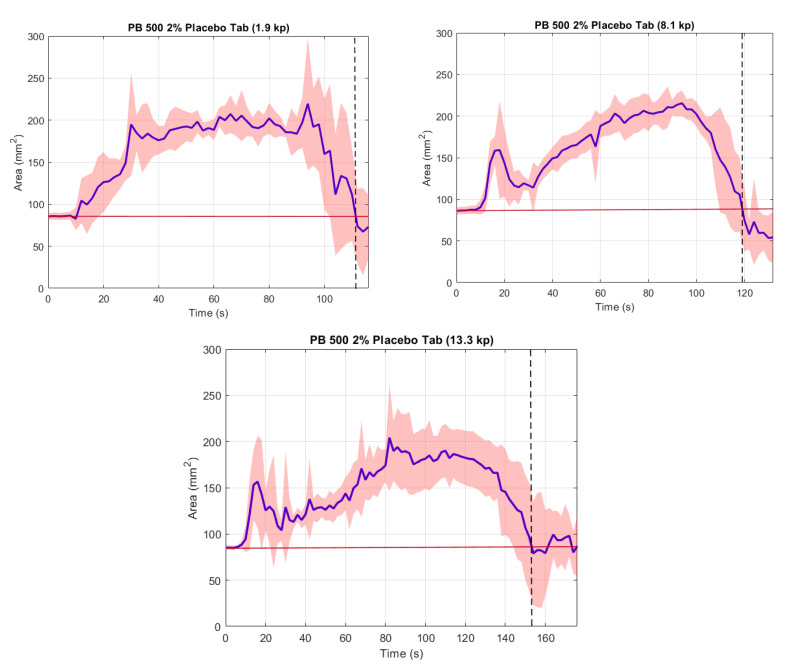
Mean top-down area-time profiles for ODTs assessed in the oral cavity model (OCM) (*n* = 6).

The ODTs were visually inspected side-on to the apparatus (Figure 3, left). In this case, the ODTs swelled, becoming wider, and eroded from the underside first (bottom up), which was in contact with the silicone tongue surface and thin film of SSF. Once concluded, a thin layer of the ODT was observed to have remained attached to the palate, which was washed away. The thickness of this layer was not recorded. By contrast, the video frames measured ODT area from a top-down view (Figure 3, right). All ODTs disintegrated similarly. The initial breakdown was at the ODT surface, while the inner tablet remained intact, which resulted in rapid ODT area growth (Figure 4). Saturation of the ODTs was followed by removal of ODT disintegrate products through co-action of saliva and compression exerted onto the dosage form by the model’s artificial tongue. As the outer region was ‘washed away’ following each compression, there was a marginal decline in the size of the area. With the ODT eroded back to a size similar to the starting area (Figure 3, lower red objects), a thin film remained, which required varying numbers of compressions to wash away the final layer.

The findings displayed in Figure 3 show that, to some extent, all ODTs adhered to the acrylic palate during testing and, afterwards, when complete disintegration occurred following visual inspection. Despite adhering to the palate, the ODTs were exposed to the thin film of SSF on the tongue surface with the saliva flowing down in the anterior to posterior direction. As testing duration and number of compressions performed increased, there was a notable increase in ODT spread across the acrylic palate. There remains the possibility that the central mass, which remained at the end of testing, may have been the central cores of the ODTs. These were, however, deemed to be swallow-able since food science studies have reported that particles size <2 mm will not injure the upper digestive mucosa. Thus, this is considered safe for swallowing [31]. The mean top-down areas over time are illustrated for all three ODTs in Figure 4. Disintegration time was determined by the return of the ODT area to the area at t = 0—the intersect between the red-solid and black-dashed lines. The reasons for choosing this endpoint were as follows.

1. The later time is assumed to correspond to ODT thin film formation and contact with the palate—as per the observation made during the experimental procedure (Figure 3, left).
2. The very small areas became erratic and deviations became larger, particularly on thin layer formation, as this would break up, becoming difficult to determine/measure using edge detection.
3. Provision of a consistent measure for a trend seen for all three ODTs.

In all instances, there was a sufficient ODT area remaining (Figure 4) after the measured endpoint. This may be explained by the numerous particles formed during the process of disintegration, which varies in size and culminates along the edges of the silicone tongue prior to being ‘washed’ away. The size of any remaining particles at the observed endpoint may be aversive to some individuals, despite little to no visibly identifiable fragments remaining in the oral cavity. Assessment of these ODTs in vivo may yield potential correlation.

A previous study examined the mouthfeel of ODTs following in vivo assessment [32] with a median particle size of the manufactured ODTs measuring 522 µm. The palatability study yielded good acceptance of the ODT formulation in participants. Another study reported that granule size within ODT formulations of up to 244 µm was acceptable [33]. Both studies were conducted in adult volunteers and used formulations with median particle sizes far larger than Pharmaburst 500 (130 µm) [34]. Thus, this demonstrates the likely acceptance of the ODTs examined within this study were in vivo mouthfeel assessments to be conducted.

As expected, an increase in ODT hardness increased disintegration time for both methods (Table 1). Differences in disintegration times for 1.9 kp and 8.1 kp were not significantly different in the OCM. However, they were more significant for the European Pharmacopoeia method. Reasons may include the closer interactions of particles and these particles becoming more tightly bound during ODT formation by compression (directly related to hardness). Visual inspection of disintegration in European Pharmacopoeia defined apparatus and manual operation of equipment. The relative volumes of fluid used in each of the methods may provide a further explanation. These are also applicable to disintegration times compared between 1.9 kp and 13.3 kp, where an increase of 36.6% for the OCM, but 209.1% with European Pharmacopoeia testing was seen.

There is, perhaps, a hardness limit beyond which porosity is below a level whereby the relatively low volumes of fluid used in the OCM method results in slower ingress and, hence, slower disintegration. The more autonomous approach with the OCM demonstrates its importance further, since, beyond a certain point, increasing hardness will not have detrimental effects on disintegration times and this is likely to be mimicked in vivo. These findings may also point to a hardness resolution limit of the OCM method, which would need to be explored further.

A previous study compared ODT disintegration with an alternate in vitro methodology to gold-standard pharmacopeia and in vivo testing [35]. When examining Pharmaburst ODTs at varying hardness, similar trends in disintegration time were observed for the two in vitro methodologies for low and medium hardness (equivalent to 8.1 kp and 13.3 kp reported here).

On closer examination of the disintegration time profile generated for 13.3 kp (Figure 4, bottom), the ODT area appears to extend above the designated endpoint beyond the intersection. This phenomenon was the result of one sample from the six analysed disintegrating values beyond the mean time reported in Table 1. Therefore, when plotted as a mean disintegration time profile, the anomaly can be observed.

The OCM may provide a more physiologically relevant estimate of in vivo disintegration profiles. The accelerated testing conditions of the European Pharmacopoeia method expose all ODT surfaces to continuous fluid movement while the OCM method ensures that, like the human oral cavity, only a single large surface is exposed to SSF. Furthermore, drawing a direct comparison with other in vitro techniques for measuring disintegration time might not be appropriate because the measurement end points differ considerably [16].

A key challenge when designing high drug-loaded ODTs is the change in in vivo disintegration behaviour, since many applications for ODT use have highly dosed drugs (e.g., paracetamol and ibuprofen), which result in longer disintegration times. Future work would aim to determine if there is any difference in disintegration mechanisms of placebo and active ODT formulations.

The key advantage presented within this pilot study using the in vitro OCM is the opportunity to ascertain disintegration behaviour profiles of ODT samples by evaluating changes in the observable area during simulated oral processing. If the unresolved questions on how to provide greater accuracy when measuring disintegration in relation to area can be answered, through further investigations and modifications of the OCM, then the findings presented will be of greater significance.

## 8. Conclusions

Three ODTs of varying hardnesses were tested by two disintegration methodologies. When examined using the established European Pharmacopoeia method, all ODT types disintegrated completely—far within the prescribed time limit of three minutes. The artificial oral cavity model was further adapted and piloted to evaluate in vitro disintegration-time profiles of ODTs, and, with the accompanying image analysis method developed in this study, present a distinct advantage in demonstrating the mechanism of ODT disintegration. By contrast to the European Pharmacopeia, less variability was observed with the oral cavity model, albeit longer disintegration times being noted. This is a reflection of physiologically relevant estimations of in vivo disintegration by exposing only a single large surface to salivary fluid, as seen in the human oral cavity. The model could have the potential to be implemented as a decision-support tool during the early stages of the drug design process to improve acceptability and further understand ODT disintegration behaviour.

## Figures and Tables

**Table 1:** Orodispersible tablets (ODT) oral cavity model (OCM) and European Pharmacopoeia (EuPh) mean disintegration times (*n* = 6).

ODT Hardness (kp)	OCM Disintegration Time (s)		EuPh Disintegration Time (s)	
	Mean	SD	Mean	SD
1.9	112	10.4	11	0.7
8.1	119	10.6	25	1.6
13.3	153	10.8	34	2.7
